# Long-time Response with Ado-trastuzumab Emtansine in a Recurrent Metastatic Breast Cancer

**DOI:** 10.7759/cureus.6036

**Published:** 2019-10-30

**Authors:** Sukesh Manthri, Sakshi Singal, Bahaaeldin Youssef, Kanishka Chakraborty

**Affiliations:** 1 Medical Oncology, East Tennessee State University, Johnson City, USA; 2 Pathology, East Tennessee State University, Johnson City, USA

**Keywords:** metastatic breast cancer, her2 positive breast cancer, ado-trastuzumab emtansine

## Abstract

Breast cancer is the most common cancer in a woman with a five-year survival of patients with metastatic disease is estimated at 23%. Ado-trastuzumab emtansine (T-DM1) is a HER2-antibody drug conjugate currently approved for the treatment of HER2-positive pre-treated metastatic breast cancer (BC). We report a case of recurrent metastatic breast cancer with unusually lengthy progression-free survival (PFS) on T-DM1 chemotherapy. She was diagnosed with Triple Positive Stage IIIC multifocal invasive ductal carcinoma of the left breast. After completing neoadjuvant chemotherapy, she underwent a bilateral mastectomy. Final pathology showed partial response. Postoperatively, she received adjuvant chemotherapy and radiation therapy. She was started on Q21 days trastuzumab following completion of adjuvant chemotherapy. Systemic imaging showed liver lesions and biopsy confirmed recurrence. She was started on T-DM1, endocrine therapy with anastrozole was continued. She is currently status post 45 cycles. T-DM1 was approved for the treatment (single-agent) of HER2-positive, metastatic BC based on phase III data from the EMILIA and TH3RESA study. Median PFS in the T-DM1 arm was 9.6 months. Herein, we present a case of a woman with recurrent triple positive metastatic BC with a lengthy progression-free survival on T-DM1 chemotherapy.

## Introduction

Breast cancer is the most common cancer in women worldwide. Amplification of human epidermal growth factor receptor 2 (HER2+) occurs in approximately 20% of breast cancers [1]. Breast cancers expressing HER2+ have higher rates of proliferation and a worse prognosis than non-HER2-expressing tumors without target therapy. The five-year survival of patients with metastatic disease is approximately 23%. Combining HER2-targeted agents with standard chemotherapy is an effective therapeutic approach for patients with HER2-positive metastatic breast cancer. Ado-trastuzumab emtansine (T-DM1) is a HER2-antibody drug conjugate which incorporates the HER2 targeted actions of trastuzumab with the microtubule inhibitor emtansine (DM1 - a maytansine derivative), resulting in cell cycle arrest and apoptosis. T-DM1 allows intracellular drug delivery specifically to HER2-overexpressing cells, thereby improving the therapeutic index and minimizing exposure of normal tissue. Currently, T-DM1 was approved by the Food and Drug Association for the treatment of HER2-positive pre-treated metastatic breast cancer.

## Case presentation

**Figure 1:**
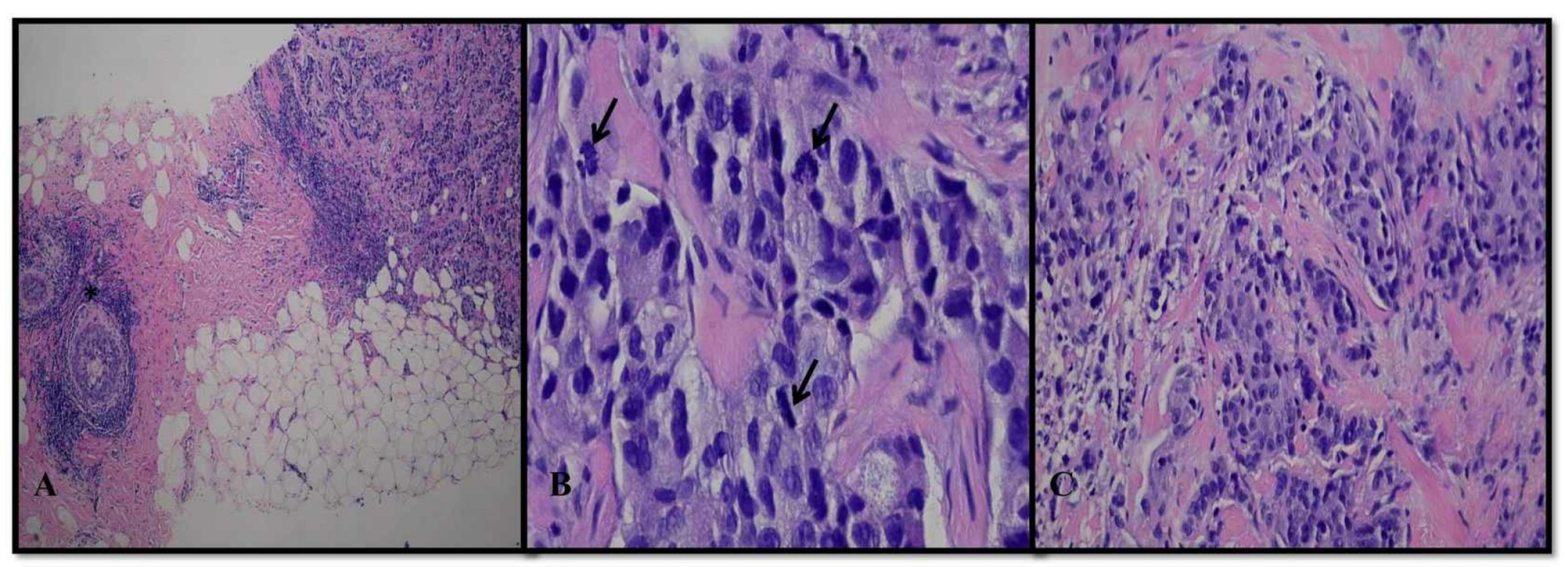
H&E stain of a left breast lesion at 40x magnification showing a poorly differentiated tumor infiltrating breast parenchyma with adjacent foci of ductal carcinoma in situ (asterisk) (A). At higher, 400x magnification the tumor is composed of sheets of enlarged cells with hyperchromatic, pleomorphic nuclei and multiple mitotic figures (arrows) (B). A second left breast lesion at 200x magnification, displays similar histopathologic features consistent with multifocal high grade invasive ductal carcinoma (C). H&E: Hematoxylin and eosin

We report a case of a 53-year-old female who initially noted a lump in her left breast. She had undergone multiple mammograms, however, most recent mammography described evidence of multiple masses. Ultrasound described a 4 x 3.2 cm irregular mass at the six o’clock position of the left breast and a second mass measuring 1.4 x 2.1 cm at the seven o’clock position and a third measuring 1.6 cm in greatest dimension. Core needle biopsies revealed Nottingham Grade 3 invasive ductal carcinoma, which was estrogen receptor positive (11-50%), progesterone positive (1-10%), and HER-2/amplified by FISH (Figure 1).

**Figure 2:**
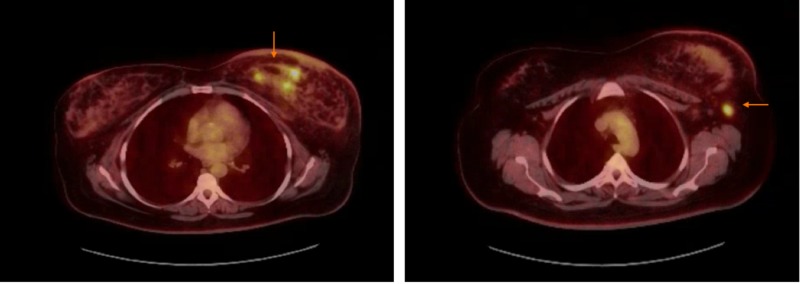
PET-CT showing multiple areas of increased radiotracer activity in the inferior left breast with a maximum SUV of 7.73 and lymph nodes in the left axilla, largest measures 1.4 x 1.0 cm. This lymph node has a maximum SUV of 6.41. SUV: Standardized uptake value

Bilateral breast MRIs showed multicentric disease in the left breast. Other areas of clumped non-mass enhancement were also concerning for surrounding ductal carcinoma in situ (DCIS). There was adenopathy on the left involving intramammary lymph nodes, left axillary lymph nodes concerning for metastatic adenopathy. Subsequently, PET-CT was done, and it showed findings consistent with malignancy in the left breast with metastatic disease to left axilla (Figure 2).

An MRI of the brain was negative with no evidence of metastatic disease. She underwent a biopsy of an enlarged left axillary lymph node and this was pathologically positive for metastatic invasive ductal carcinoma. She was diagnosed with Triple Positive Stage IIIC multifocal invasive ductal carcinoma of the left breast. She was initially treated with neoadjuvant chemotherapy based on data from the neo-sphere trial with four cycles of docetaxel, trastuzumab, and pertuzumab, and later she underwent left modified radical mastectomy along with prophylactic right simple mastectomy.

Final pathology showed partial response. It showed a reduction in tumor burden and sizes of foci were 2.0 cm and 2.5 cm; 5/6 lymph nodes were positive for histological grade 3. Post-operatively course was complicated by left mastectomy flap necrosis status post debridement and wound vacuum-assisted closure (VAC) placement. Due to poor wound healing, adjuvant chemotherapy and radiation treatments were delayed. She received four cycles of adjuvant chemotherapy with dose-dense adriamycin, Cytoxan and 45 Gy (25 fractions of 1.8 Gy daily) of adjuvant radiation therapy directed to the left chest wall, the left supraclavicular nodes, and the left axillary lymph nodes. She was started on Q21 days trastuzumab following completion of adjuvant chemotherapy, and adjuvant endocrine therapy with anastrozole was initiated after completion of radiation therapy.

**Figure 3:**
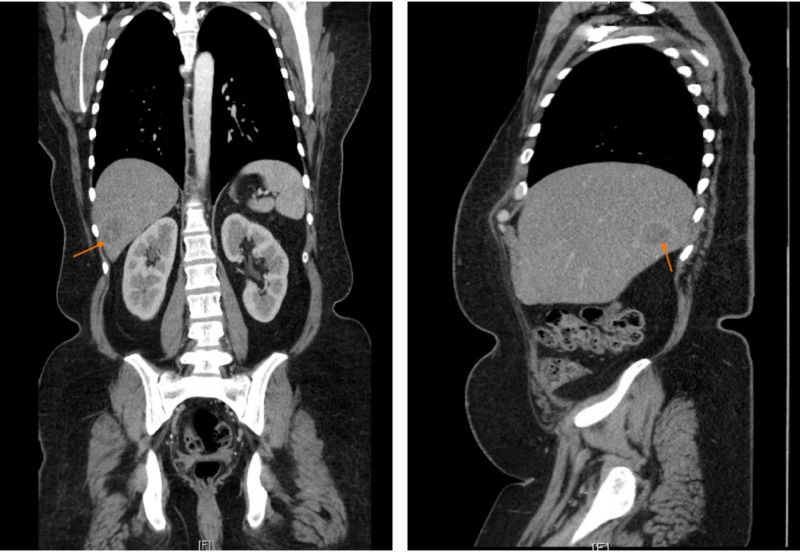
CT chest/abdomen/pelvis with contrast showing large mass seen in the right lobe of liver measuring 3 cm x 2.5 cm suggestive of metastasis.

Computerized tomography (CT) chest/abdomen/pelvis done due to incomplete response to neoadjuvant treatment, showed a large mass in the right lobe of liver measuring 3 cm x 2.5 cm and was suggestive of metastasis (Figure 3).

**Figure 4:**
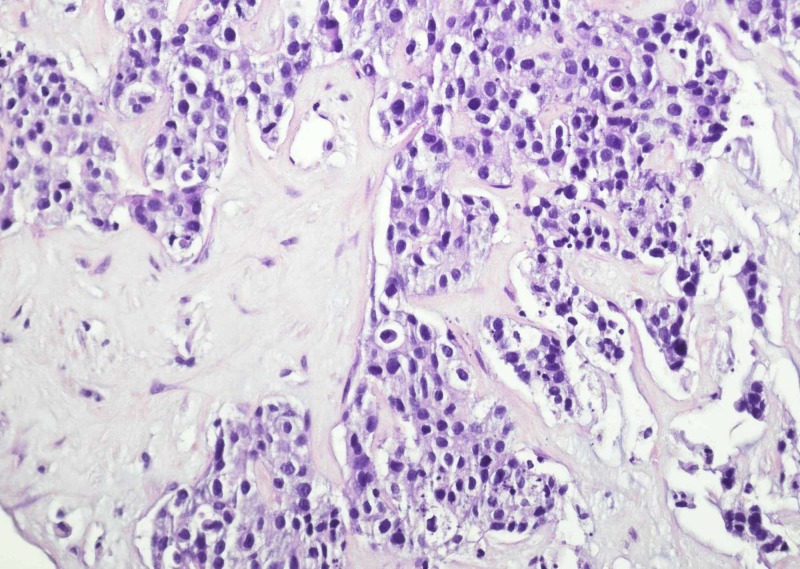
Liver biopsy is remarkable for infiltrating nests of tumor.

Liver biopsy revealed metastatic adenocarcinoma with immunostains consistent with breast primary (Figure 4).

At that point, she was started on T-DM1, endocrine therapy with anastrozole was continued. She was initially receiving T-DM1 Q21 days, subsequently, due to grade 1 fatigue and thrombocytopenia, it was changed to Q28 days cycle.

She is currently status post 45 cycles (20 cycles of Q21 days and 25 cycles of Q28 days) [2]. Her ejection fraction remained stable at 60-65%.

## Discussion

Ado-trastuzumab emtansine (T-DM1) is a HER2-antibody drug conjugate which incorporates the HER2 targeted actions of trastuzumab with the microtubule inhibitor emtansine (DM1 - a maytansine derivative), resulting in cell cycle arrest and apoptosis. Once T‑DM1 binds to the HER2 receptor, it allows intracellular drug delivery specifically to HER2-overexpressing cells, consequently improving the therapeutic index of the drug and minimizing its exposure to normal tissue [3]. T-DM1 was approved in 2013 by the U.S. Food and Drug Administration (FDA) for treatment (single-agent) of HER2-positive, metastatic breast cancer based on data from the EMILIA trial [4]. In this trial, T-DM1 demonstrated improved median progression-free survival (PFS) compared with capecitabine and lapatinib (9.6 vs. 6.4 months, respectively; p < 0.001). Median OS also favored T‑DM1 (30.9 vs. 25.1 months, respectively; p < 0.001). Overall response rate and duration of response were also superior in patients treated with T-DM1 [4].

Another randomized phase III trial, TH3RESA study, compared T-DM1 to the treatment of the physician’s choice in a patient who progressed on two or more anti-HER2 treatments. PFS was significantly improved with trastuzumab emtansine compared with physician's choice (median 6.2 months vs. 3.3 months; p < 0.0001) [5]. Overall survival was significantly longer with trastuzumab emtansine versus treatment of physician's choice (median 22.7 months vs. 15.8 months; p = 0.0007) [6]. The incidence of grade 3 or worse adverse events occurred in 40% of patients in the trastuzumab emtansine group. Most common grade 3 or worse adverse events were thrombocytopenia (6%) and hemorrhage of any type (4%). Our patient developed grade 1 fatigue and grade 1 thrombocytopenia during her treatment course.

The drug (T-DM1) is given as an intravenous infusion every three weeks (21-day cycle) until disease progression or unacceptable toxicity occurs. DM1 is metabolized by CYP3A4 and CYP3A5 in liver microsomes. With a dose of 3.6 mg/kg, the terminal half-life (t1/2) is 3.5 days [7]. It has a linear pharmacokinetic process and preclinical trials show that T-DM1 catabolites are eliminated by the biliary system with minimal participation of the renal system [8]. For patients with toxicities, first dose reduction to 3 mg/kg and second dose reduction to 2.4 mg/kg is suggested. Usually for grade 3 or 4 thrombocytopenia (platelets 25,000/mm^3^ to <50,000/mm^3^), withholding treatment until platelet count recovery to ≤ grade 1 (platelets ≥75,000/mm^3^) is suggested. Our patient was initially receiving T-DM1 Q21 days for 20 cycles, subsequently due to grade 1 fatigue, thrombocytopenia and to ensure better tolerability it was changed to Q28 days cycle. She is currently status post 45 cycles (20 cycles of Q21 days and 25 cycles of Q28 days). Her ejection fraction remained stable at 60-65%.

## Conclusions

Historically T-DM1 was studied in who progressed on two or more anti-HER2 treatments and PFS was approximately 6.2 to 9.6 months. To our knowledge, no such cases with lengthy progression-free survival on T-DM1 chemotherapy in metastatic setting were reported in the literature. It is currently unclear if a specific subset of HER2 positive breast cancer patients gains maximal benefit. Identification of predictive biomarkers and the concurrent development of diagnostics for these biomarkers are needed. Herein, we present a case of a woman with recurrent triple positive metastatic breast carcinoma with such an unusually lengthy progression-free survival on T-DM1 chemotherapy.
